# Editorial Notes

**Published:** 1907-06

**Authors:** 


### Editorial Notes.

**Dual Hospital Appointments.**

THE question as to whether it is or is not desirable that physicians or surgeons should have the option of holding office at more than one hospital has recently been the subject of discussion in Bristol, the committee of one of the largest medical charities having been opposed to the dual appointment of a member of their staff.

In this city we are perhaps especially fortunate in that our great medical charities are watched over and provided for by laymen who have devoted such an amount of time and money to the work they have taken in hand, that no one can doubt for one moment their single-hearted desire for the best interests of their respective institutions.

In approaching this or any question which concerns a great hospital, we must bear in mind that these institutions are really great public trusts, which exist, not for the benefit of medical practitioners, but in the first instance for the welfare of the patients who go to them for treatment, and secondly, for the advancement and teaching of medicine in all its branches; but it is obvious that the latter is in reality one of the essential factors in promoting the interests of the patients in any hospital.

The honorary staff of the particular hospital in question have pointed out that it is desirable that members of the staff "should be encouraged to practise as pure physicians and surgeons, because in that case they have more time to devote to hospital work, and therefore greater opportunities for the attainment of special knowledge and skill; and the charge of beds is an additional inducement to assistant-physicians and surgeons to adopt this line of practice." With this view we entirely agree, and would further add that we consider that those who hold appointments in the chief hospitals in a medical centre which desires and expects a University status, ought to devote themselves to medicine, surgery, or one of the special branches, and not to engage in general practice.

It may prove desirable and necessary to lessen the number of physicians and surgeons at a hospital in order to provide sufficient scope for their acquiring the necessary experience in a limited field of practice ; but it is obviously to the best interests of any hospital to have a few masters of medical or surgical practice, rather than a larger number of general practitioners.

To leave it open to any physician or surgeon of a hospital to engage in any or every line of practice for gain, but to lay it down that the only restriction on his freedom is that he may not devote his spare time to acquiring further experience in his particular branch of medicine or surgery, not for gain but for pure love of, and keenness in, his profession, is surely to " strain at a gnat and swallow a camel."

Nothing redounds more to the credit of a hospital than to find that its physicians and surgeons are sought after, not only by the rich patients in the surrounding district, but by the smaller hospitals for the poor attending them, and we feel that it is only natural and right that the small hospitals should desire a " slice off the rich man's cake " for their own patients' benefit.

We have not touched on the question from the point of view of the physicians and surgeons on a hospital staff. But there is surely some right on their side to determine how far their liberty to employ their spare time shall be restricted by the charities they serve. If it is suggested that by serving more than one hospital any individual physician or surgeon might be overloading his time and energies, the same may be said of a large private practice, and it would be as reasonable to curtail the time he occupied in serving his private patients, or even acting in any public capacity, as to dictate limits to his services to the poor.

It is quite possible that there is room for improvement in hospital management, both in our own city and elsewhere, but any alterations must be carefully guided in the right direction, and any question that arises approached in an open spirit, not by the Governors only, but by the doctors also, who are partners, not servants. Indeed, it is the reputation and enthusiasm of individual members of the staff on which the fame and status of any hospital mainly depends.

We have heard it said that the medical staff gain much from the prestige of their appointments; but although here in Bristol, as elsewhere, it is a privilege to be associated with a great hospital, any prestige arising therefrom is an asset created and maintained by medical men, not by the philanthropists to whom Bristol owes so much for providing the no less, but no more, essential buildings and necessary funds.

It is not, as some seem to imagine, the hospital which makes the man, but the man who makes the hospital, although in doing so he may bring credit to himself. To cite but a single example from the many names which have made Bristol hospitals renowned, we may point to Greig Smith, who made our Royal Infirmary known throughout the civilised world by his surgical work there. Yet how empty is all this reflected honour, for unless the individual man, like Greig Smith, gives far more than he takes, a hospital appointment proves for him of little or no value whatever; it is only a missed opportunity.

**The Cossham Memorial Hospital.**

THE opening of this new hospital is an event which must prove of considerable importance to the district in which it is situated. The site is an ideal one, and the buildings placed upon it have been constructed with every possible care to make them as ideal as the grounds.

Arrangements are being made for fifty-two beds, and it is not intended that there shall be any out-patients. Every care is to be taken that the privileges and duties of the local medical men shall not be infringed. As the hospital is well endowed, there will be no need for any appeals to the philanthropy of the public; there will be no subscribers, and therefore no subscribers' letters of recommendation. With adequate funds for the purpose, it is to be hoped that the services of the working staff shall in time receive due remuneration.

We give on another page an outline of the history and arrangements of the buildings and the constitution of the medical and resident staff.

The elevated position of the hospital, its isolation from any near buildings, its graceful style of architecture (early Georgian), and its central cupola with illuminated clock and Westminster chimes, will cause this hospital to become a prominent landmark, embellishing the whole district.

